# Effect evaluation of a heated ambulance mattress-prototype on thermal comfort and patients’ temperatures in prehospital emergency care – an intervention study

**DOI:** 10.3402/ijch.v74.28878

**Published:** 2015-09-14

**Authors:** Jonas Aléx, Stig Karlsson, Ulf Björnstig, Britt-Inger Saveman

**Affiliations:** 1 Department of Nursing, Umeå University, Umea, Sweden; 2 Artic Research Centre, Umeå University, Umea, Sweden; 3 Center for Disaster Medicine, Unit of Surgery, Department of Surgery and Perioperative Science, Umeå University, Umea, Sweden; 4 Center for Disaster Medicine, Umeå University, Umea, Sweden

**Keywords:** thermal comfort, thermal discomfort, finger temperature, cold exposure, Cold Discomfort Scale, cold stress, active heat, heat transfer

## Abstract

**Background:**

The ambulance milieu does not offer good thermal comfort to patients during the cold Swedish winters. Patients’ exposure to cold temperatures combined with a cold ambulance mattress seems to be the major factor leading to an overall sensation of discomfort. There is little research on the effect of active heat delivered from underneath in ambulance care. Therefore, the aim of this study was to evaluate the effect of an electrically heated ambulance mattress-prototype on thermal comfort and patients’ temperatures in the prehospital emergency care.

**Methods:**

A quantitative intervention study on ambulance care was conducted in the north of Sweden. The ambulance used for the intervention group (n=30) was equipped with an electrically heated mattress on the regular ambulance stretcher whereas for the control group (n=30) no active heat was provided on the stretcher. Outcome variables were measured as thermal comfort on the Cold Discomfort Scale (CDS), subjective comments on cold experiences, and finger, ear and air temperatures.

**Results:**

Thermal comfort, measured by CDS, improved during the ambulance transport to the emergency department in the intervention group (p=0.001) but decreased in the control group (p=0.014). A significant higher proportion (57%) of the control group rated the stretcher as cold to lie down compared to the intervention group (3%, p<0.001). At arrival, finger, ear and compartment air temperature showed no statistical significant difference between groups. Mean transport time was approximately 15 minutes.

**Conclusions:**

The use of active heat from underneath increases the patients’ thermal comfort and may prevent the negative consequences of cold stress.

During prehospital emergency care, especially in geographical areas with sub-arctic climates as in northern Sweden, it is common that patients are exposed to low temperatures with accompanied cold stress. Cold stress aggravates the medical condition, pain perception and anxiety (1–3). Further, in an earlier study we have shown that the milieu in ambulances during these circumstances may be too cold to offer good thermal comfort for the patients; verified by, for example, decreasing finger temperature during transport (4). Being cold is also experienced as an uncomfortable subjective experience. Despite this, exposure to cold temperatures is often a neglected problem in prehospital care (5). The recommendations and guidelines that currently exist regarding the type of products and materials to be used for purposes of preventing and treating cold exposure are usually based on local traditions.

When the core body temperature drops, the body starts producing heat by shivering in an attempt to mitigate decreasing body temperature. Shivering is maximal at 35°C core body temperature and disappears when the temperature goes below 33°C. Shivering causes major discomfort to patients (6), and patients experience frustration at not being able to stop cold-induced, uncontrolled body movements. In an earlier study, shivering has been regarded as one of the worst experiences when injured in a cold environment (7). Among many adverse effects, shivering leads to increased stress on blood circulation and can be dangerous, for example, to older people with compromised circulation (8). Particularly vulnerable groups in need of special attention are the very young and old patients who have an impaired ability to protect themselves from hypothermia, as well as those with diseases reducing the body's natural cold response (9).

Active warming can be supplied by, for example, chemical heating pads, hot air blankets and electric blankets, whereas passive rewarming is based on insulation from external cold and wind, as well as on reducing heat loss from the body by using, for example, blankets (10,11). There are a number of studies (1,2,12–15) describing both passive and active methods. Active warming is mostly recommended in prehospital care (13,16). Active heat placed on top of the patient (air, blankets or chemical pads) has been shown to have a positive impact on patients’ thermal comfort (4,13,17) and anxiety (12,17,18).

Reducing patients’ exposure to cold, preserving body heat and preventing a decreasing core temperature are therefore important treatments in the prehospital care (10,19,20). In ambulances in Sweden, it is uncommon to use active warming and the reason is often limited to protecting the patient from further heat loss by using blankets and sheets, often with low insulation value (19). To the best of our knowledge, there is a lack of research about experiences of thermal comfort and active heat supplied from underneath for ill and injured prehospital patients. In a quasi-experimental study on healthy students using active heat supply from underneath, we have shown an improved thermal comfort with this method (21). Therefore, the aim of the present study was to evaluate the effect of an electrically heated ambulance mattress-prototype on thermal comfort and patients’ temperatures in the prehospital emergency care.

## Method

### Design

A quantitative intervention study.

### Setting

Data were collected in Västerbotten County Council in the north of Sweden. The outdoor temperature during the investigating months was on average +2°C.

### Participants

**Fig. 1:**
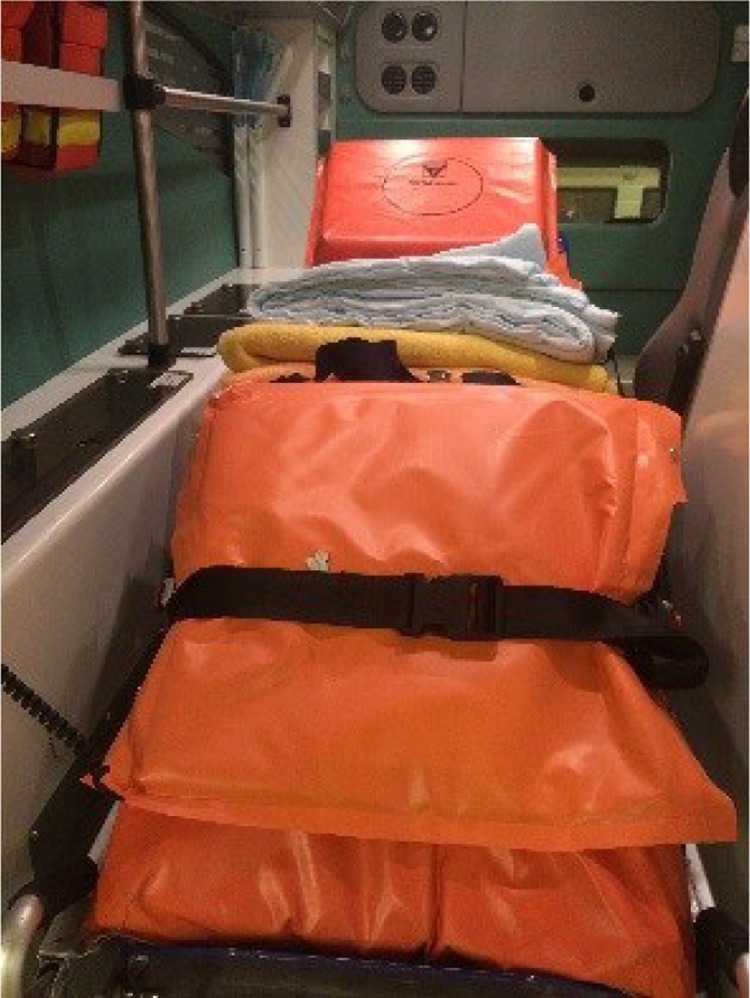
The heated mattress on the stretcher connected to 12 V electrical system.

Included were 60 patients, aged 18+ years transported by 2 ambulances, one intervention and one control. Unconscious patients, patients having communication problems and patients having severe and life-threatening injuries or illness with extensive care needs were excluded. The ambulance used for the intervention group (n=30) had an electrically heated mattress (Fig. 1) on the regular stretcher, and the stretcher in the ambulance used for the control group (n=30) had a regular ambulance stretcher without heating. Three patients in the control group and one patient in the intervention group were outdoors at arrival.

### Data collection

Data on the Cold Discomfort Scale (CDS) and of finger temperature were measured and documented on 3 occasions, (a) when arriving to the patient, (b) after 10 minutes on the stretcher and (c) at arrival to the emergency department (ED). CDS is a subjective judgment scale for the assessment of experienced thermal state, ranging from 0 to 10, where 0 indicates not being cold at all and 10 indicates unbearable cold. It is a sensitive and validated scale (22).

An infrared thermometer measured the finger temperature with dual laser points indicating the measurement area, (CIR8819). Measurements were taken approximately 7 cm from the measurement surface (3.5 cm Ø). Accuracy: ±1°C or ±2%. Resolution: 0.1°C. The Ear tympanic temperature was measured by using an infrared light thermometer (Braun Thermo Scan, Exac temp IRT 8520, Germany) after 10 minutes in the ambulance compartment. Accuracy: ±0.2°C (35.5–42°C). Resolution: 0.1°C. The ambulance compartment temperature was measured after 10 minutes by an extern sensor (Bead probe 6030) connected to the infrared thermometer (CIR8819). When the participants had been lying on the stretcher for 10 minutes, they were asked if they experienced the stretcher to be warm or cold when they initially lay down and now how they experienced it.

### Procedure/intervention

All ambulance staff working in the two ambulances were well informed of the study, the purpose and the procedure before the study started.

Data were collected during approximately 15 days in November and December 2011 and the same amount of days in November and December 2014, only during daytime.

The first author and 2 ambulance nurses collected the data. For each participant, the study started when the ambulance staff arrived to them and lasted until the patient arrived to the ED. The ambulance transport mean time was approximately 15 minutes.

The patients were asked to participate and were informed that they could withdraw their participation at any time without explaining why and that it would not affect their care. The patients then gave verbal-informed consent. For the intervention, a 150-cm-long electrical ambulance mattress-prototype was used on the regular ambulance stretcher. The heated mattress was connected to the 12 V electrical system in the ambulance and generated approximately +35°C surface temperature of 50W and was not to be regulated (Fig. 1). The heated mattress was on constantly, that is when the ambulance had no current assignment and during ambulance transport. It had the same texture as the regular mattress and did not affect the softness. Participants in both groups lay on a cotton sheet and were covered with a polyester blanket that is standard in ambulance care in Västerbotten.

### Analysis

The sample size calculation showed that at least 22 patients were required in each group. A difference in mean score of CDS rating between the group receiving care on a heated mattress (intervention) and the group (control) with an unheated mattress was estimated to be 1.5. Standard deviation was estimated at 2.0 (cf. 21) with a power of 80% and a significance level of 5%.

Difference in mean, significance level and standard deviation were calculated. Independent sample t-test was used for normally distributed data, whereas Chi-square test, Mann–Whitney U test and Friedman test were used for non-parametric data. The statistical analyses were performed with IBM SPSS software (version 21 SPSS Inc., Chicago, IL, USA).

### Ethical consideration

The study was approved by the Regional Ethical Review Board in Umeå (2011-343-31M). Nobody outside the research team had access to the data. All collected data were treated confidentially. All results were analysed on a group level making it impossible to identify individual participants.

## Results

**Fig. 2:**
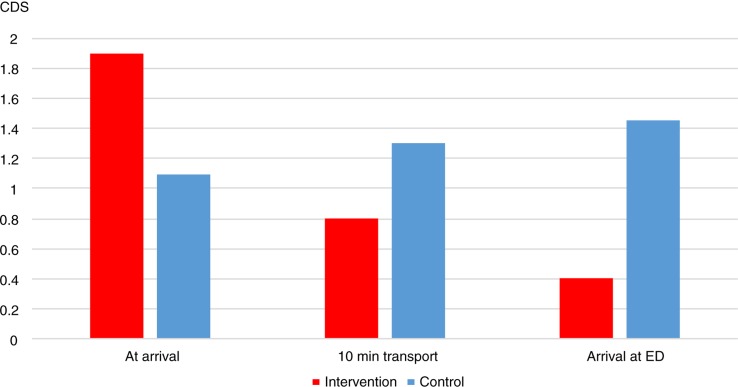
CDS measurement for intervention and control group at arrival to the patient, after 10 minutes transport and at arrival to the ED. Friedman test: Intervention (p<0.001), Control (p=0.014).

The background data and the patients’ reason for calling an ambulance are shown in Table I. In the intervention group, the rating of thermal comfort (CDS) improved during the ambulance care compared to a decreased rating in the control group (Fig. 2). On arrival to the patients, there were no significant differences between the intervention and the control group regarding CDS rating score and finger temperature concerning ear temperature or air temperature in the ambulance compartments (Table II).

**Table I:** Background data and patients’ reasons for requesting ambulance care

	Intervention n=30	Control n=30	p
Age (mean, SD)	76.4 (16.7)	68.3 (16.7)	0.11^a^
Sex	(n, %)	(n, %)	0.30^b^
Men	14 (47)	18 (60)	
Women	16 (53)	12 (40)	
Reasons for requesting			
Respiratory insufficiency	3 (10)	2 (7)	
Cardiovascular symptoms	10 (33)	12 (40)	
Severe illness	5 (17)	4 (13)	
Trauma	4 (13)	5 (17)	
Pain	5 (17)	2 (7)	
Fainting	2 (7)	2 (7)	
Abdominal pain	1 (3)	3 (10)	

a Mann–Whitney U test

b Chi-square test.

**Table II:** Similarities between the intervention group and the control group at arrival and of the compartment temperatures after 10 minutes transport

	Intervention group (n=30)			Control group (n=30)			
			
	Min	Max	Mean (SD)	Min	Max	Mean (SD)	p
CDS	0	8	1.9 (2.4)	0	6	1.1 (1.9)	0.67^a^
Ear temperature	36.0	37.8	36.9 (0.4)	35.4	40.0	36.9 (0.9)	0.15^a^
Finger temperature	16.8	35.1	28.8 (4.6)	14.4	36.6	27.7 (4.8)	0.39^b^
Compartment temperature	16.7	29.3	21.5 (3.3)	13.2	25	20.1 (3.1)	0.14^b^

a Mann–Whitney U Test

b independent sample t-test.

A significant higher proportion of the patients in the control group (57%) rated the regular stretcher as initially cold to lie down on compared to the patients in the intervention group (3%, p<0.001). There was only a small difference, not statistically significant, regarding the participants’ rating of the back as warm, after 10 minutes in the ambulance; 100% in the intervention group and 93% in the control group rated their back as warm (p=0.492). The heated mattress had a positive impact on how the patients rated the scores on the CDS at the first measurement. The difference in CDS ratings between the first measurement at arrival to the patient and the last measurement at arrival to the ED differed significantly between the groups (p<0.001; Table III and Fig. 2). However, there was no impact on finger temperature between intervention and control group (Table III).

**Table III:** Difference in mean of CDS and finger temperature between the first measurement at arrival to the patient and the last measurement at arrival to the ED

	Intervention (n=30)	Control (n=30)	p
Diff CDS^a^ (mean, SD)	−0.93 (0.50)	1.43 (0.51)	<0.001
Diff Finger^b^ (mean, SD)	−4.2 (3.25)	0.88 (2.98)	0.57

a Mann–Whitney U test

b independent t-test.

## Discussion

The present study shows that using a heated mattress that supplies the patients with active heat during ambulance care improved the thermal comfort, in comparison with the use of the regular mattress which instead aggravated thermal discomfort. Therefore, these results are in line with data from other studies with different active systems (13,14,17). However, there are no other studies showing an increased thermal discomfort during ambulance transport on a regular stretcher.

In the present study, a much higher proportion of patients in the control group experienced the regular stretcher as initially uncomfortably cold compared to those in the intervention group. This experience is in line with an earlier study, in which 90 participants were divided into an intervention group receiving active heat and a control group that received passive heat placed on top of the patients. Almost all who received active heat estimated it as comfortable while most who did not receive active heat had a less positive experience (12).

Heat flows spontaneously from a hot body to cold. If the environment is cooler than the body, the body loses heat to the surroundings (23). When patients lay down, for example, on a cold mattress (cooler than body temperature), the body heats up the cold surface by losing heat through conduction (9,19). In the present study, almost all participants in both groups rated their back as warm after 10 minutes, indicating that patients in the control group had transferred heat to the cold mattress. A patient lying down on an already heated mattress loses less energy through conduction, and this saved energy may be important for the vulnerably ill and injured patients and could potentially prevent increased morbidity and even mortality.

There were no significant differences regarding finger temperature when lying on a stretcher supplied with a heated mattress or not. A possible reason might be that hand temperature is affected by many different factors. The short transport time might be a main factor as to why no difference was observed between groups in this study. After vasoconstriction is initiated, skin temperature rises relatively slowly when returning to a warm condition compared to how fast the skin temperature falls in cold environments (24). Thus, a transport of approximately 15 minutes may not have been long enough for vasodilatation to initiate. However, it is optimal to monitor skin temperature gradients at sites such as fingers (25). It is, therefore, not possible to further comment on the reason why no increase in finger temperature was seen in the intervention group.

In emergency situations, especially in cold climates, it is important to avoid chilling when energy loss involves discomfort. A previous study has shown that patients lying on the cold ground felt cool within few minutes (7). After a while, the cold discomfort worsened and quickly became the patients’ primary concern, regardless of their injuries. The regular ambulance stretcher has shown to adapt quickly to the cold surrounding environment in wintertime, therefore patients may have to lay down on a mattress as cold as below zero degrees (4).

The background data show some discrepancy in the patients’ ages and reasons for requesting ambulance care (Table I). The participants also had various medical conditions, which can affect the body's heat production (9). This can in turn lead to an enhanced experience of feeling cold, which of course might influence the results.

### Methodological considerations

The participants in this study were not homogenous which can be both a disadvantage and an advantage. The different diagnosis and medical condition may have influenced the effect regarding the heated mattress and thermal comfort; however, it is clear that the thermal comfort increased in the intervention group and decreased in the control group. Our group of participating patients gives a relevant picture of what kind of patients are cared for in prehospital emergency care. This increases the possibilities to generalize the results to other ordinary prehospital contexts such as ambulance care, mountain rescue teams and rescue teams at sea.

Tympanic temperature measurement has been shown to be a fair better estimate of the core body temperature, better than rectal temperature, when comparing to measurement with a pulmonary artery catheter which is regarded as the best indicator of core body temperature (26). Tympanic temperature has shown a very small discrepancy to oesophageal and bladder temperature measurement and is an easy, non-invasive and relevant method for core body temperature monitoring in prehospital research and ambulance care (27).

A few patients were excluded due to life threatening illnesses or injuries; meaning that the results outcome might have been different if these patients had been included.

## Clinical implication and future research

We believe that a heat supply from underneath is a basic nursing intervention to increase thermal comfort. It is easy to implement, and going forward, it would be advantageous to have ambulances equipped with an active heating mattress as standard. Further studies on device handiness and feasibility are needed for a successful large-scale implementation. More controlled intervention studies on thermal comfort, cold stress and active heated supply in the prehospital care may be of value. Further studies should preferably also include measurements of various physiological data and longer transport time.

## Conclusion

The use of active heat from underneath increases the patients’ thermal comfort and might prevent the negative consequences of cold stress.
